# Tools for the Quality Control of Pharmaceutical Heparin

**DOI:** 10.3390/medicina55100636

**Published:** 2019-09-25

**Authors:** Anthony Devlin, Courtney Mycroft-West, Patricia Procter, Lynsay Cooper, Scott Guimond, Marcelo Lima, Edwin Yates, Mark Skidmore

**Affiliations:** 1 Molecular & Structural Biosciences, School of Life Sciences, Keele University, Huxley Building, Keele, Staffordshire ST5 5BG, UK; a.devlin1@keele.ac.uk (A.D.); c.j.mycroft-west@keele.ac.uk (C.M.-W.); p.procter@keele.ac.uk (P.P.); l.c.cooper@keele.ac.uk (L.C.); m.andrade.de.lima@keele.ac.uk (M.L.); eayates@liv.ac.uk (E.Y.); 2 Institute for Science and Technology in Medicine, Keele University, Keele, Staffordshire ST5 5BG, UK; s.e.guimond@keele.ac.uk; 3 School of Biological Sciences, University of Liverpool, Crown Street, Liverpool L69 7ZB, UK

**Keywords:** heparin, glycosaminoglycans, spectroscopic methods, chemometrics, quality control, analysis

## Abstract

Heparin is a vital pharmaceutical anticoagulant drug and remains one of the few naturally sourced pharmaceutical agents used clinically. Heparin possesses a structural order with up to four levels of complexity. These levels are subject to change based on the animal or even tissue sources that they are extracted from, while higher levels are believed to be entirely dynamic and a product of their surrounding environments, including bound proteins and associated cations. In 2008, heparin sources were subject to a major contamination with a deadly compound—an over-sulphated chondroitin sulphate polysaccharide—that resulted in excess of 100 deaths within North America alone. In consideration of this, an arsenal of methods to screen for heparin contamination have been applied, based primarily on the detection of over-sulphated chondroitin sulphate. The targeted nature of these screening methods, for this specific contaminant, may leave contamination by other entities poorly protected against, but novel approaches, including library-based chemometric analysis in concert with a variety of spectroscopic methods, could be of great importance in combating future, potential threats.

## 1. Introduction

The anticoagulant drug heparin is one of the oldest drugs to date in clinical use and is the second most widely used pharmaceutical drug by mass [1]. A naturally occurring carbohydrate, it is a member of the glycosaminoglycan (GAG) family of linear, polydisperse polysaccharides that consist of alternating amino-sugars and uronic acids [2]. Heparin chains contain motifs that bind with high affinity to antithrombin (AT), allowing the inhibition of both factor Xa and thrombin, and thereby the common pathway in the coagulation cascade [3,4]. Of the motifs responsible for binding to antithrombin, the most studied is that of the pentasaccharide sequence, which has been made synthetically as the oligosaccharide, fondaparinux sodium (Arixtra®) [5]. Fondaparinux, along with other types of low molecular weight heparin (LMWH) drugs, primarily inhibit factor Xa and are used therapeutically for their improved pharmacokinetics and reduced side effects over heparin [6]. Heparin activity is not limited solely to antithrombin and both are known to bind and regulate many proteins.

Heparin is found exclusively in the granules of mast cells, where it protects and enhances the activities of serine proteases during degranulation; although the physiological significance of this is not currently well understood and the true physiological role of heparin remains, to date, unknown. [7]. Heparin and HS are associated with numerous disease states owing to their ability to bind and regulate a multitude of distinct molecules, including histamine, cytokines (e.g., interleukins (IL) 2 through 4), chemokines (e.g., IL-8 and PF4), growth factors (e.g., transforming growth factor beta (TGFbeta), fibroblast growth factors (FGFs), vascular endothelial growth factor and hepatocyte growth factor/scatter factor) and selectins (e.g., L- and P-selectin). Heparin and HS are also associated with the protein aggregation of amyloid-beta and tau-proteins, which have been implicated in Alzheimer’s disease. Furthermore, heparin and HS interact with numerous pathogens, including the hepatitis B and C, human papilloma and human immunodeficiency viruses and the malaria parasite. A number of protein binding features within heparin and HS have been proposed and the nature of these interactions with the aforementioned proteins have been reviewed extensively in [7]. 

## 2. Heparin Structure

The fine structure of heparin remains, to date, unknown and the mechanisms that underpin biosynthesis remain poorly understood [8,9]. Heparin biosynthesis is not template driven and distinct classes of biosynthetic enzymes do not always modify the polysaccharide chain to completion [8]. The polydisperse and heterogeneous nature of heparin consequently presents a significant challenge for the pharmaceutical quality control of this biomolecule.

**Figure 1:**
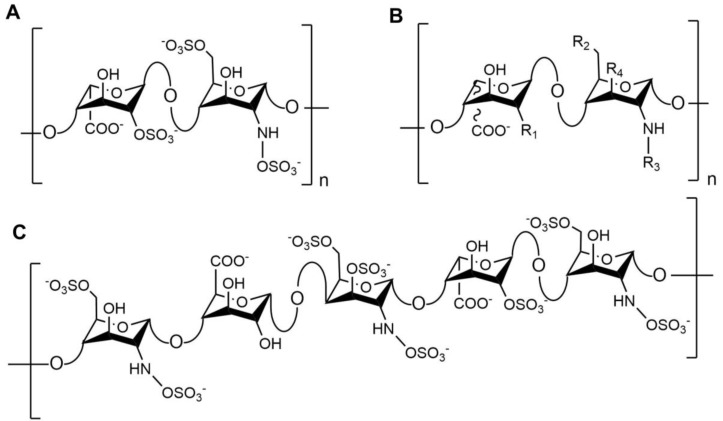
Structure of heparin. (**A**) The most common disaccharide repeat found in heparin, Ido2S-GlcNS,6S. (**B**) The extent of heparin disaccharide structures, where R 1, R2 and more rarely, R4, may be O-sulphated (OS) or -OH and R3 may be either acetyl, sulphate or rarely a free amino, and the uronic acid may be either β-**D**-glucuronic acid (GlcA) or α-**L**-iduronic acid (IdoA). (**C**) Pentasaccharide sequence associated with antithrombin binding. The initial residue may be N-sulphated or N-acetylated.

**Figure 2:**
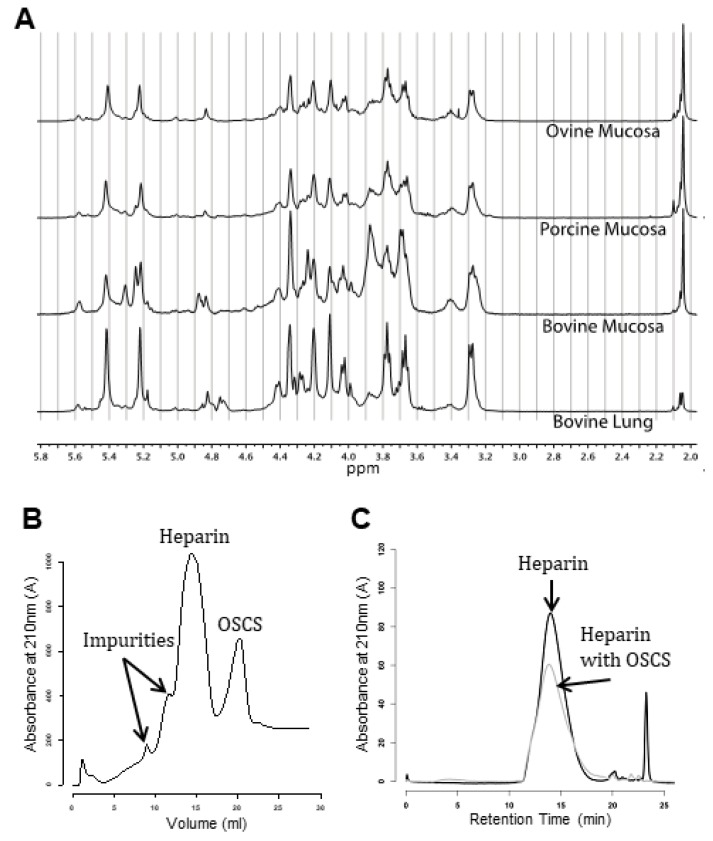
Examples of the current methods used for heparin quality control. (**A**) Proton nuclear magnetic resonance (NMR). (**B**) Strong anion exchange high-performance liquid chromatography (SAX-HPLC chromatogram). (**C**) Size exclusion chromatogram showing molecular weight distribution profile. OSCS: over-sulphated chondroitin sulphate.

The structure of heparin possesses increasing levels of complexity from monosaccharide composition, through to higher orders of structure. At its simplest level, heparin is a linear chain of repeating amino (α-D-glucosamine (GlcN)) and uronic acid (either β- D-glucuronic acid (GlcA) or α- L-iduronic acid (IdoA)) monosaccharide building blocks, which may be variably sulphated. The heparin backbone encompasses a disaccharide repeating unit of GlcN with either GlcA or IdoA. GlcN may be unmodified (amine, NH_2_), N-sulphated (NS) or N-acetylated (NAc) and O-sulphated at position C_6_ and more rarely at position C_3_ (6OS and 3OS, respectively) the uronate residues may be O-sulphated at position C_2_ (2OS). Theoretically, 48 disaccharides are possible (Figure 1B), but usually fewer are observed within mammalian heparin samples, the levels of which may be probed for structural analysis (Figure 1). Typically, in excess of 70% of constituent disaccharides of heparin are tri-sulphated and 70–80% are typically Ido2S-GlcNS,6S (Figure 1A), with the remaining 20–30% consisting of the other less common disaccharides [2,10,11,12]. The average level of sulphation, for all disaccharide units that comprise the polysaccharide chain, is often expressed as the degree of sulphation (DoS) and this can be used as an indicator of the underlying structure of heparin, however it offers little indication regarding the complexity of the higher-level structure. The underlying structure of heparin is known to be tissue specific and differs between species, with individual animals of the same species exhibiting differences. Such variations are observed throughout the molecular weight populations, disaccharide compositions, and anticoagulant activities (Table 1, Figure 2A) [1,10,13,14,15] 

An additional level of heparin complexity and that of its sister molecule heparan sulphate (HS) encompass charge heterogeneity across the chain and are the result of linked biosynthesis. Unlike heparin, HS possesses a domain structure with distinct charge densities that are a result of varying disaccharide constituents. Within HS there are three domains: the NA-domain, which is primarily un-sulphated; the mixed (NA/NS) domain, which contains un-sulphated IdoA; and the NS-domain, which contains primarily IdoA2S-GlcNS, all of which may contain 6OS [11,26]. The high content of Ido2S-GlcNS6S within heparin prevents combinatorial domain structure from forming, however there are motifs dispersed throughout the chain that can bind to other biomolecules. The discovery of a pentasaccharide sequence (containing a rare 3OS modification) consisting of GlcNAc/NS6S-GlcA-GlcNS3S-IdoA2S-GlcNS6S (Figure 1C) affords heparin the ability to bind with high affinity to antithrombin and inactivate factor Xa, while a longer oligosaccharide sequence facilitates binding to antithrombin and sequestration of thrombin (factor IIa), resulting in a subsequent anticoagulant effect [27].

The flexibility of the pyranose rings, the ability to rotate about constituent glycosidic linkage regions, and the ability to form intramolecular hydrogen bonds affords heparin a further level of complexity. Association with cations alter the conformations and bond angles depend on the size and charge of the associated cation molecule [28]. Heparin has been modelled as exhibiting a right-handed helix with a pitch of around four residues [29,30,31] but, this interpretation depends on making some simplifying assumptions to enable interpretation of the NMR data [29]. IdoA residues have been shown to adopt ^1^C_4_, ^4^C_1_, and ^2^S_0_ ring conformers, which interchange dynamically with minimal alteration to the glycosidic bond conformation [31,32,33]. The 2OS of IdoA and the presence of NAc on the neighbouring glucosamine is thought to favour adoption of the ^2^S_0_ conformation, in part due to a hydrogen bond between the two, stabilising the conformation [31] opposed to GlcA and GlcN which exist almost entirely in the rigid ^4^C_1_ form [34]. The absolute arrangement of atoms in a three-dimensional space is driven by the aforementioned features of the heparin chain [35,36] and as a result it is not currently possible determine the 3D structure for an entire heparin chain [37]. This high level of complexity affords heparin the ability to interact with many distinct biomolecules, the effects of which propagate beyond the binding region, for example upon antithrombin binding, the entire population of IdoA that is in the ^2^S_0_ configuration within the heparin chain increases, despite the fact that not all of iduronates are involved directly in antithrombin binding. This is thought to enable tighter binding at the binding site, while in heparin: FGF-1 and -2 binding, the ^1^C_4_ conformer population is favoured, as 6OS and not IdoA2S is relevant to FGF binding [33].

Heparin coordinates readily with cations owing to its high negative charge, with the native cation forms of heparin present within a given physiological or pathological system largely unknown. It is assumed to be a mixture of Na^+^ and Ca^2+^, due to their high molarities in serum, however K^+^ ions may bind cooperatively with Na^+^ [28]. Cations appear to stabilise the ring structures permeating heparin into specific forms, homogenising bond-angles and conformer populations, resulting in altered conformational states across the molecule. For pharmaceutical use heparin is prepared in Na+ and Ca2+ forms for parenteral administration, but Li+ form may also be utilised as coatings for medical devices. Aside from these, many different cation forms of heparin have been studied [28,38,39]. Cations with higher charge intuitively bind with higher affinity to heparin [28], but atomic radii regardless of charge may impact the ability to coordinate with heparin.

Potentially, a higher additional level of heparin structure, whereby interactions between distinct heparin chains could occur. While there would appear to have been little/no direct studies to date of this phenomenon, the fact that heparins interact with many other molecules, coordinate with cations, and form intramolecular hydrogen-bonds, the potential exists for heparin to form intermolecular hydrogen bonds, share coordination with cations, and ultimately possess this level of structural complexity.

As described above, the structural complexity of heparin is incredibly high, and therefore quality control is very difficult. All levels of a heparin structure can and do affect spectral and activity data; a perfectly reasonable pharmaceutical heparin may simply be in a strange cation form and therefore exhibit unusual activities or spectral features. Further compounding this, there exists no way to fully sequence heparin chains and thus intuitively discover molecule binding motifs of interest [40]. Furthermore, binding motifs that are known (such as the pentasaccharide sequence) require wholesale changes of the heparin chain to bind tightly, the ramifications of which are not fully understood [12]. Heparin synthesis is also relatively misunderstood: the enzymes and the steps they catalyse are well documented, but the reasons they do not go to completion (producing different domains and motifs, etc.) are as yet poorly understood. To compound this, carbohydrate synthesis is not template-driven and can alter depending on the conditions and status of the parent cell. As such, man-made, synthetic heparin that is comparable to natural heparin is currently unviable. Therefore, pharmaceutical heparin is a natural product, usually obtained from porcine or bovine intestine, but can be sourced from ovine intestine and bovine lung as well; however, owing to bovine spongiform encephalopathy (BSE) in the late 1980s and early 1990s, heparin from porcine intestinal mucosa is used predominantly in Europe and North America. This means that commercially sourced heparin (for drug markets or research) is naturally heterogeneous and was therefore regulated and controlled primarily based on its relative biological activity, not by its structural composition [41].

## 3. Original Screening Techniques for Pharmaceutical-Grade Heparin

Early monographs were aimed at establishing whether a given sample possessed the same anticoagulant activity as a known control, hence, heparin quality control was primarily focused on assaying for biological activity, assuming that anything with comparable anticoagulation potential of sufficient potency was acceptable as pharmaceutical-grade heparin. A simple clotting test (the activated clotting time (ACT)) was prescribed, although this approach essentially overlooked the subtleties and complexity of heparin and proved easy to circumvent with alternative entities [42,43].

## 4. Contamination of Pharmaceutical Heparin, 2007–2008

Potency as a screening method possessed a fatal flaw; it assumed that the heparin being assayed was pure heparin and that a contaminated sample would possess a lower potency and therefore fail the screen. The limitations of this assumption were exposed in 2007–2008 when batches of heparin demonstrated severe, adverse side effects, resembling the symptoms of anaphylaxis, which were later established to be dual activation of the contact system [44,45]. This resulted in greater than 100 fatalities in North America alone. Identification of the contaminant proved a considerable challenge, but through a concerted effort of numerous laboratories, utilising nuclear magnetic resonance (NMR), capillary electrophoresis (CE), and optical rotation, a contaminant possessing a related structure to heparin, a high sulphation level and anticoagulant activity was discovered: that of an over-sulphated chondroitin sulphate (OSCS) [41,46]. In this instance, the OSCS was produced by sulphate augmentation upon a chondroitin sulphate precursor, leading to an over-sulphated (sulphated at every available hydroxyl position) chondroitin sulphate with an average DoS of four. Due to the unusually high number of associated side-effects, it was posited that more than one contaminant was present in pharmaceutical heparin. It was proposed that other GAGs that may be present during the crude extraction process may also have been over-sulphated and formulated with the final crude products thereby possessing an unnaturally high DoS, albeit with a similar anticoagulant effect to that of “pure” heparin, however this has not been confirmed [45].

## 5. Changes to the Heparin Monograph Post-Contamination

Following the heparin contamination, new techniques aimed at confirming the identity of heparin prior to potency screening were made mandatory, resulting in the addition of two techniques to the heparin United States pharmacopeia (USP) monograph and to the monographs of many other pharmacopoeias namely 300 MHz ^1^H NMR spectroscopy [47] and capillary electrophoresis (CE) [48,49,50] 

^1^H NMR offers a high-resolution technique that can demonstrate the presence of contaminants. Most, if not all, of the major heparin ^1^H NMR signals have been assigned with 16 characteristic peaks routinely used to confirm the presence of heparin in the sample. [51,52]. Changes to, or a lack of, any of these peaks may indicate a contaminant or a suboptimal heparin sample. Due to the high charge density of heparin and other GAGs, they are amenable to electrophoretic migration and separation when placed within an electric field [43,53]. Separation by capillary electrophoresis may be considered similar to that of the reversed-phase high-performance liquid chromatography (HPLC) technique, but through use of narrow capillaries, allows for very small sample quantities to be separated. As OSCS (or any over-sulphated, GAG contaminant) has a larger DoS compared to heparin, it will migrate in a distinct manner within the electric field and thus can be separated [49]. Both techniques can detect OSCS contamination at the level of 0.5% and 1%, respectively [49,54,55] and have been introduced into the USP monograph. NMR and CE are orthogonal techniques that elucidate different components within the structure of heparin. Hence, features of heparin that may be overlooked in one technique will be observed within the other [56,57]. For example, unusual sequences that resist enzymatic digestion will be poorly resolved or ignored entirely in separation techniques but may be detected by NMR [58], thereby providing a more robust form of quality control. 

Since the addition of NMR and CE in 2008, the monograph has been updated [48], prescribing a higher field strength, that of a 500 MHz NMR spectrometer. This increases the spectral resolution of the spectrum and therefore its discriminatory power. Strong anion exchange high-performance liquid chromatography (SAX-HPLC) has replaced CE on the monograph as this technique affords improved separation of potential contaminants, is more universally available, requires less-specialised training, and does not suffer from reproducibility issues that have been shown to plague CE [51]. Size definition of the heparin chains has also been incorporated to promote an additional measure of heparin consistency, with acceptable heparin possessing no more than 20% of its population exceeding 24,000 Da; an average molecular weight (MW) between 15,000 and 19,000 Da and a ratio of populations between 8000: 16,000 and 16,000:24,000 Da of no less than 1.0 [6,52]. The upper limit exists to diminish potential side effects associated with higher MW larger chains [59], while the average exists to ensure good potency and the ratio serves to prevent blending of failed heparin lots with accepted ones [6]. It is important to note however, that side effects associated with larger heparins have only been seen when comparing full-length heparin to LMWH and may not be due entirely to chain length [6]. Examples of these techniques, including both *bona fide* heparin and contaminated samples can be found in Figure 2. The clotting assays were also revised, opting for the AT: factor Xa and AT: factor II chromogenic assay, which have a higher specificity for heparin bioactivity. 

## 6. Alternative Techniques that May Assist in Heparin Quality Assurance

Alongside the techniques employed within the heparin monograph, there are a multitude of other techniques that are well documented for the study of heparin that may be applied to its quality control and can be broadly separated into three orthogonal methodologies: species separation, structural elucidation, and size definition. 

### 6.1. Species Separation

Electrophoretic separation has already been used in the heparin monograph in the form of CE, where detection of OSCS and dermatan sulphate (DS) (a common impurity in heparin) at the levels of 0.05% and 0.1%, respectively, have been achieved [49,60,61,62]. Alternative electrophoretic formats such as polyacrylamide gel electrophoresis (PAGE) has also been used to distinguish OSCS from heparin post nitrous acid treatment, a procedure that affects NS groups found in heparin but not OSCS, allowing for their separation [55]. PAGE separation using this method relies on the contaminant to not be N-sulphated, and thus may not be used when prior knowledge of the contaminant is unknown, however it has detected OSCS contamination at the level of 0.1%, whilst being low-cost and facile for performance in most labs [63]. Agarose gel electrophoresis has also been applied, with 1% OSCS detection reported [53,64]. 

Chromatographic methods are in use for 42nd heparin monograph in the form of SAX-HPLC, but there are numerous alternatives that may be of use, as reviewed in [22]. Retention by SAX is based on competition between the analyte with counter-ions in the mobile phase, whereby analytes with small charge/size ratios will elute first. Weak anion exchange (WAX)-HPLC, where the competitive counter-ions are part of the stationary phase, has also been applied to heparin quality control. SAX-HPLC can detect 0.02%–0.1% OSCS [22,51] while WAX-HPLC can detect OSCS at the level of 0.5%, with questionable detection at 0.25% [65]. SAX-HPLC still remains the monograph-approved option, however WAX-HPLC provides the ability to trap OSCS oligosaccharides on the column, redirecting heparin to waste-allowing multiple injections of the same sample to reveal low OSCS contents [63]. Reversed-phase ion-pair (RPIP)-HPLC has also been used, where ion-pairing reagents are introduced to the mobile phase, modifying the retention of a charged analyte to a hydrophobic stationary phase. RPIP-HPLC is particularly suited to saccharide analyses of samples, where individual di-, tri-, and tetra saccharide levels can be determined. Due to the unnatural sulphation of OSCS, it resists digestion by conventional enzymes and therefore does not depolymerise into its saccharide constituents, allowing easy detection and resulting in reduced peak integrals on RPIP chromatograms. This effect is unique, however to OSCS, and other more GAG-like contaminants or impurities will be digestible, but their constituent saccharides can then be detected. Hydrophilic interaction chromatography (HILIC) where oligosaccharides are separated based on their overall polarity has also been used and can separate 1% OSCS contamination, with an estimated lower level at 0.2% [66,67]. Chromatographic methods are useful in quality control as they can separate many possible contaminants, assuming they can be separated by the desired method. Unusual chromatograms may be indicative of novel contaminants and hence they can be used proactively. These techniques suffer however when separating contaminants or impurities with similar primary structures, as they may have the same underlying structural features. 

Methods of separation lend themselves to further coupling in the form of downstream detection methods, such as mass spectrometry (MS) [63,68], fluorescent detection [53,69], and additional size definition [70]. MS methods can be employed downstream, post-depolymerisation and separation by other means [71,72] but they may also be employed to separate whole heparin molecules and other GAGs [73]. Multiple ionization techniques have been utilised [74]. Generally, depolymerisation performed as part of the MS ionisation procedure offers challenges, as S–O bonds are more labile than glycosidic bonds, nevertheless the oligosaccharide fragments created by these techniques are relatively well documented, hence unknown or unusual fragments, corresponding presumably to contaminants can be identified and quantified accordingly, whilst also facilitating a higher throughput [73,75]. Performing hyphenated separations using established methods allows an increase to the sensitivity of the individual methodologies, for example HILIC and RPIP-HPLC separation coupled to MS has detected 0.1% and 0.5% OSCS contamination [76,77]. 

MS is expensive and technically challenging when compared to the methods it is hyphenated downstream of, and so other methods to increase the sensitivity of the initial separation strategies have been explored, typically involving the labelling of depolymerised saccharides, to allow for increased sensitivity of detection [78]. Typically, 2-aminoacridone (AMAC) has been used as it affords significantly increased detection compared to that of UV. Other fluorescent tags have been utilised, such as 4,4-Difluoro-5,7-Dimethyl-4-Bora-3a-4a-Diaza-s-Indacene-3-Propionic Acid, Hydrazide), to tag and detect heparin disaccharides, expansion of which to other GAG species is possible and would allow for detection of heparin and GAG contaminants [69]. Labelling techniques have been applied to chromatography, CE, PAGE, and MS, improving all of their sensitivities [66]. 

Further separation of depolymerised species is another avenue of considerable interest; impurities such as over sulphated heparan sulphate (OSHS) may respond very similarly to heparin during separation as they possess similar charge densities and disaccharides, separation of which is very difficult due to their similar charge densities, polarities, and hydrophobicity. Isoelectric focusing is one method that has been used to further separate these as described in [70]. If such a method could be coupled in a 2-dimensional manner to conventional PAGE, a very fast and cheap quality control method could be developed.

### 6.2. Structural Elucidation

While ^1^H NMR spectroscopy is a part of the heparin monograph due to its high resolution and complete peak assignment, there are many other ways that NMR spectroscopy could be utilised, for example employing ^13^C nucleus, which is able to distinguish identical chemical features located in distinct saccharide sequences [79]. ^15^N NMR has also been studied, with some nitrogen microenvironments being assigned, producing fingerprints of intact and modified heparins [80,81,82,83]. Some contaminants such as sulphated agarose or OSHS may lurk beneath the spectrum of heparin however, avoiding detection [84,85]. The true potential for NMR in quality control lies in powerful higher-dimensional experiments, including homonuclear experiments such as correlation spectroscopy (COSY) [55,86,87], total correlation spectroscopy (TOCSY) [46,80,81], heteronuclear experiments, such as heteronuclear single-quantum correlation spectroscopy (HSQC) [46,56], heteronuclear multiple-quantum correlation (HMQC) [56], through-space correlations such as nuclear-Over Hauser effect spectroscopy (NOESY) [46,88], and diffusion methods such as diffusion ordered spectroscopy (DOSY) [89]. These 2D NMR methods have been used in the study of the higher-level structure of heparins and were pivotal in first defining OSCS as a contaminant [41,46]. Furthermore, HSQC and HMQC using the ^1^H and ^13^C dimensions have been used to distinguish contaminants other than OSCS, such as OSHS which possesses a very similar overall structure to heparin, while the ^15^N dimension has been used to fingerprint heparins [80]. DOSY, which measures the differences between diffusion coefficients between molecules has also been applied to detecting OSCS contamination, showing clearly OSCS contamination and also distinguishing it from other types of contamination [89]. HSQC with SAX-HPLC have also been validated as complementary methods that may study heparin structure and therefore its quality [90]. 2D NMR was however deemed too complex, time-consuming, and expensive for use in the heparin monograph.

Circular dichroism (CD) is another form of spectroscopy, where a polarised electromagnetic wave passes through a chiral substance and is able to have its right and left circularly polarised components absorb to different extents, which is indicative of the overall topology within the studied substance. For heparin, the IdoA and GlcA residues are diastereoisomers, which absorb the polarised electromagnetic waves differently to each other, while oxygen atoms in glycosidic linkages, pyranose rings, hydroxyl groups, N-sulphates, and nitrogen atoms in NAc groups also produce spectral features in CD [34]. CD is a sensitive way to detect topological changes in heparin and has been used to study its cation derivatives and interactions with target proteins [34,38]. CD could be used to detect contamination, due to the alteration of normal heparin bands appearing in contaminated samples, with down to 3.5% OSCS contamination detected when isolating wavelengths correlated to known contaminants [91].

Vibrational spectroscopies such as infrared (IR) and Raman may also be used in heparin quality control. Bonds within molecules vibrate at characteristic frequencies, depending on the type of bond, constituent atoms, and surrounding environment, all of which culminates in a unique spectrum for each studied molecule. IR spectroscopy has previously been used to study heparin primary and secondary structures and cation forms [30,82,83]. While IR spectroscopy studies the absorption of infrared light into a molecule, Raman spectroscopy studies the release of the absorbed light at a different energy level, providing similar, yet complementary, information to IR spectroscopy. When applied to OSCS screening, 38/41 samples could be correctly identified with IR and 36/38 samples could be correctly identified with Raman spectra, down to the level of 1.3% OSCS [58]. These methods produce spectra with broad and overlapping bands, however they are further susceptible to environmental conditions.

### 6.3. Size Definition

The size definition of heparin is yet another challenging task as heparin exists as a heterogeneous population of varying MWs. Larger heparin molecules possess increased activity but provide a larger surface area for potential side effects to occur [6]. The size of heparin can be defined in many ways, including legacy methods such as ultracentrifugation and viscosity measurements [86,92] and more modern methods such as calibration through use of standards, utilising PAGE [54,87,93,94], and size exclusion chromatography (SEC) [94,95,96,97] or through use of the Rayleigh theory, whereby the ratio of scattered light intensity to incident light intensity is used to define MW, such as with static-right-angle-, low-angle- and multi-angle laser light scattering (SLS, RALS, LALS, and MALS respectively) techniques [52,88,94,98,99,100]. MW has also been defined using ^13^C NMR [101] and MS [102,103]^.^ Size definition when compared to standards is difficult, as standards are hard to define or acquire, meaning that they are usually created in-house, leading to variation between labs, however a USP MW standard is available to try to reduce this [6,14,94]. A triple detector (TDA) approach has also been utilised, where a RALS detector, a differential refractive index detector (SLS detection), and a viscometer are used in concert to accurately define the MW of heparin and may also be used to create uniform standards [104]. MW determination can also be used to screen for contaminants due to the resistance of OSCS to depolymerisation by bacterial heparinises, resulting in easy detection [105].

### 6.4. Other Methods

Other, less conventional methods have also been applied to heparin screening. Full-length heparin and OSCS competitively inhibit many DNA polymerases, including Taq polymerase, the enzyme used in PCR. Therefore, the digestion of the heparin component should leave any OSCS, and subsequently prevent the PCR from occurring. This rationale has been applied to a quick heparin screen, and as low as 0.16% OSCS contamination has been detected [106]. A microplate-based array has also been applied to heparin screening, taking the form of a colorimetric assay, which changes colour depending on the molecule (full length or depolymerised heparin and OSCS for example) bound to the cationic polythiophene polymer (3-(2-(N-(N0-methylimidazole)) ethoxy)-4-methylthiophene (LPTP)). The colorimetric shift can be recorded, and perturbations in the shift correlating to 0.001% OSCS have been reported [107]. Fluorescent methods have also been developed, typically taking the form of a Förster resonance energy transfer (FRET) system, whereby some fluorescent molecule that interacts with both heparin and OSCS is introduced to the test sample and through manipulation of the heparin molecule, either through enzymatic depolymerisation [108,109,110,111,112,113] leaving only OSCS to cause fluorescence, or cation modification [114] resulting in preferential binding of and subsequent detection of OSCS. Between 0.5% and 0.0001% OSCS contamination has been detected with these methods. The differing charge densities between heparin and OSCS have also been exploited to help detect OSCS, whereby the electric potential across an anionic-sensitive membrane can be altered depending on the charges of the GAGs present, and hence 0.5% and theoretically lower OSCS and DS can be detected [115]. These techniques, while powerful, are strictly applicable to OSCS and in some examples DS, as they rely on the unique interplay between OSCS and heparin and cannot be applied to quality control of any other or novel foreign body.

## 7. Novel Issues Facing Heparin Quality Control

While numerous techniques exist that may be used or applied to the quality control of heparin, none of them directly combat one of the major issues in heparin analysis: its high levels of heterogeneity. As there currently exists no heparin standard that is completely sequenced, has a uniform molecular weight distribution, and cation form, it is very difficult to confirm the fidelity of each technique to the samples true structure. However, pharmaceutical heparin has been in circulation since 1935 and has caused little upset during this time, albeit for the contamination in 2008. In 2011, Rudd et al. [116] put forward the idea of screening for a proposed heparin against a library of previously accepted heparins and through use of the chemometric method principal component analysis (PCA). The authors were able to create a region in the N-dimensional space where accepted heparins should fall, and thus detect “unusual” heparin samples (i.e., those contaminated with OSCS down to the level of 3%).

Chemometric analyses are data-based methods of information extraction that break down complex data sets into a series of comparable trends. There are many chemometric analyses that may be applied to these libraries, which may be split broadly into two styles: trained and untrained. Trained analyses, such as partial least squares regression (PLS), K-means clustering (KMC), and machine learning algorithms, require a priori knowledge of acceptable groupings and subsequently form models that can be used to interrogate unknown samples for their likelihood to fall into the pre-described groups, for example the groups “contaminated with OSCS” and “uncontaminated or pure heparin” could be formed and tested against. Untrained techniques, such as PCA and hierarchical clustering, require no groupings or input from the analyst, but they may not be as powerful at discriminating samples with known differences. Trained methods are applicable to known contaminants and can be used to increase the sensitivity of library screening techniques, assuming there are a series of samples in the library with known levels of the contaminant, while untrained techniques are more suited to general screening, simply showing if samples are different or similar on the basis of the information provided.

Library-based methods however, are limited by the spectroscopy they are applied to; with NMR, samples such as OS-agarose sulphate (OSAS) or OS-dextran sulphate (OSDxS) possess spectra that lie beneath the heparin spectra and so are poorly detected to the level of ~10% for each [116]. Different spectroscopic techniques have been applied to library-based chemometric methods, including IR spectra [85] which was able to distinguish OSCS contamination down to 1% and OSAS and DxS down to 5% and 2.5%, respectively and MS, which could potentially distinguish 0.1% OSCS contamination [73,117]. UV spectroscopy has also been used with PCA, to detect as little as 0.1% contamination with OSCS [118]. Trained methods offer higher sensitivity: through use of machine learning, lower OSCS contaminations and levels of DS impurities can be detected with ^1^H NMR spectra, down to 1% for both [119], while through use of PLS, 38/41 and 36/38 samples were correctly identified down to the level of 1.3% OSCS contamination as pure or not with IR and with Raman spectra, respectively [58].

Heparin is not limited to contamination by other molecules, its very nature means that it may be contaminated by itself. Heparins from other animal species or tissues possess different structures and activities (Table 1), discrimination of which is very difficult with current techniques, especially methods that separate by disaccharides, as all heparin contains the same primary constituents, with differing secondary structures. This is particularly pertinent in modern times, as some countries do not use heparins from different animal sources for religious or previous medical issues such as BSE. Library-based chemometric techniques have been applied to this too; HSQC spectral features associated with as little as 1% bovine or ovine heparin content in porcine samples have been uncovered through interrogation of PCA loading plots [116], and spectral distinction between the two has been shown with both HSQC [120], HMQC [121] and IR, as well as potential discrimination down to 10% bovine contaminated with porcine in IR [85]. Heparin species detection lends itself particularly well to trained chemometric techniques, as distinct and well-defined groups are formed based on where the sample was acquired from, however there seem to be few studies currently which address this.

One of the major risk factors for heparin contamination lies in its early stages of production. Typically, the porcine intestine is collected and processed in slaughterhouses approved by regulatory authorities before transportation to crude heparin processing facilities, where the mucosa is removed and, following several steps, a highly anionic GAG mixture is extracted. The mixture is filtered, precipitated, and vacuum dried to form the substance formally known as crude heparin—a mixture, typically comprising approximately two parts heparins and one part “other” substances, including other unwanted GAGs such as CS, DS, or HS [41,122]. While the final steps that create pharmaceutical quality heparin from crude heparin are performed under current good manufacturing practice (cGMP) regulations, the traceability of the porcine intestine suppliers that create the crude heparin is poor and, since multiple sites are used, the risk of contamination is greatly increased. There is no minimum standard for a crude heparin, so manufacturers may wish to screen crude heparins prior to processing. Crude samples have been studied for potential OSCS content using the same techniques required by the monograph and they were shown able to detect 1%–10% OSCS [123]. Library-based chemometric methods have also been applied to compositional analysis of crude heparins, grouping samples by their impurities and levels of structural features using NMR [122], but not as of yet by levels of OSCS contamination.

2D spectral approaches, using the approach of Noda et al., may also be of interest in the future of contamination detection. There are two methods of 2D spectroscopy: homo- and hetero-spectroscopic. Hetero-spectroscopic 2D analysis is when spectra are recorded following some perturbation introduced by the analyst and regions of the spectrum are assigned to these perturbations. The perturbations may be global, following a change in temperature or pH, or more targeted, exciting specific molecular environments and watching how they interact with neighbouring environments as in nuclear Overhauser effect spectroscopy (NOESY) experiments, giving detailed information as to the 3D structures of heparin. Hetero-spectroscopic 2D approaches may also be utilised, whereby a synchronous and an asynchronous spectrum of two different spectroscopic methods are calculated and can be used to visualise how these spectra relate to one another. This has been applied to IR and CD spectra to better understand the effects of Cu^2+^ cation binding [124]. Information regarding the 3D structure of heparin may be helpful for future heparin screening, as features hidden within spectra or unusual interactions or conformations that may be introduced by OS—variants of similar GAGs are currently undetectable or poorly—detectable by other means have the potential for detection.

The power of 2D spectroscopy is highlighted with NMR [46,56,80,81,84,89,116] where multiple approaches have been utilised to significantly increase its sensitivity. The complex nature of 2D-spectroscopy calculation and interpretation however has meant that such methods are not used for the heparin monograph. If chemometric or other applications are applied to 2D analyses, they may serve to reduce the complexity greatly, allowing for much easier interpretation and their potential introduction to the monograph.

## 8. The Future of Pharmaceutical Heparin

Ideally, pharmaceutical quality heparin would be chemically synthesised, however the tools and technology to do so are currently not yet available. Hexa- and dodecasaccharides serving as mimetics for LMWHs, not full-length heparin, have been produced and examined as potential therapeutics for Alzheimer’s disease [125]. Sulphated chain lengths higher than this are currently challenging to obtain [126]. To overcome this hurdle chemo-enzymatic synthesis has been deployed, whereby a chemically-synthesised precursor has been modified utilising the heparin/HS biosynthesis enzymes. Furthermore, 3OS oligosaccharides have been chemoenzymatically synthesised and a subset of these have biological activities comparable to natural heparin [126,127,128]. Both synthetic and chemoenzymatic routes remove many of the associated issues with heparin quality control as a model heparin molecule can be synthesised to allow for improved quality control, as well as removal of the associated socioeconomic issues related with the use of animal-derived products. Unfortunately, the costs and time required for these syntheses proves unviable for the current market, nevertheless synthetic and chemoenzymatic approaches are an important consideration for the future of pharmaceutical heparin. 

## 9. Conclusions

The future of heparin quality control is uncertain, the current established methods work to prevent previous issues that have arisen but seem particularly focused on defining and preventing contamination from particular molecules such as OSCS. The incredibly complex structure of heparin makes it difficult to screen as there are no sequenced heparin standards to compare methods to. Owing to this, chemometric methods have become more important and the idea of a heparin library to compare test samples against is well established in the literature [73,84,129]. There are numerous methods that can investigate heparin quality, all of which lend themselves to library creation and subsequent analysis. Powerful techniques such as 2D NMR spectroscopy are deemed too complex for the heparin monograph despite their unparalleled ability to distinguish contaminants. Chemometric methods may be used to combat this, due in part to their outputs which are easy to manage and understand, furthermore, trained methods allow for highly sensitive detection of known contaminants and impurities. Chemometric methods do not give a simple yes/no output however, analysts still need to inspect outputs and make subjective conclusions based on their discretion, an area in further need of research. Application of these methods to crude heparins is also important, as the raw starting material of heparin may be easily contaminated due to crude heparin manufacturers disperse nature. Finally, the need for such methods of quality control could be drastically reduced or even totally removed if a viable, entirely synthetic heparin were able to be produced.

## Figures and Tables

**Table 1:** Structural and biological activity of heparins extracted from distinct species, based on published results [2,15,16,17,18,19,20,21,22,23,24,25]. Data are expressed as “minimum–maximum (mean)”.

	Ido2S-GlcNS,6S	Specific Activity IU/mg			Mw/Da
		Anti-Xa	Anti-IIa	APTT	
**Porcine Mucosal Heparin**	51.5–85 (68.3)	145–220 (194)	172–230 (197)	145-277 (196)	12,000–27,090 (19,002)
**Bovine Lung Heparin**	70–87 (79.8)	105–156 (133)	130.6–180 (153)	89–167 (139)	12,000–15,240 (14,230)
**Bovine Mucosal Heparin**	47.4–64.2 (54.5)	113.6–159 (134)	92.2–160.7 (126)	88.1–181 (136)	14,900–16,417 (15,439)
**Ovine Mucosal Heparin**	60–89.4 (75.2)	196–205 (201)	191–201 (195)	165–165 (165)	12,200–20,023 (14,773)
